# Diabetes and Its Link with Cancer: Providing the Fuel and Spark to Launch an Aggressive Growth Regime

**DOI:** 10.1155/2015/390863

**Published:** 2015-04-15

**Authors:** Sanket Joshi, Menghan Liu, Nigel Turner

**Affiliations:** Department of Pharmacology, School of Medical Sciences, UNSW Australia, Kensington, NSW 2052, Australia

## Abstract

Diabetes is a disease involving metabolic derangements in multiple organs. While the spectrum of diabetic complications has been known for years, recent evidence suggests that diabetes could also contribute to the initiation and propagation of certain cancers. The mechanism(s) underlying this relationship are not completely resolved but likely involve changes in hormone and nutrient levels, as well as activation of inflammatory and stress-related pathways. Interestingly, some of the drugs used clinically to treat diabetes also appear to have antitumour effects, further highlighting the interaction between these two conditions. In this contribution we review recent literature on this emerging relationship and explore the potential mechanisms that may promote cancer in diabetic patients.

## 1. Introduction

Type 2 diabetes (T2D) and cancer are two of the most prevalent diseases facing modern society. Recent estimates suggest that close to 400 million people worldwide have T2D [1], while 12.7 million cancer cases and 7.6 million cancer deaths are reported each year [2]. Both diseases are multifactorial in origin and cancer is recognized as being a particularly heterogeneous disease.

Both T2D and cancer are characterized by marked alterations in metabolic profile and recent epidemiological evidence suggests a close link between diabetes and some forms of cancer [3]. Indeed, individuals with diabetes have significantly higher likelihood of developing a range of different cancers including liver, pancreatic, colorectal, breast, endometrial, and bladder cancers [4, 5]. The molecular basis for this link has not been fully elucidated but likely relates to changes in several factors, including nutrient availability and growth factor signaling. In this review we will briefly describe the metabolic alterations that are present in T2D and cancer and will discuss some of the factors that may potentially link these two diseases. We will also examine emerging evidence around therapeutic agents that may have utility in treating aspects of both diseases.

## 2. Metabolic Features of Type 2 Diabetes

**Figure 1:**
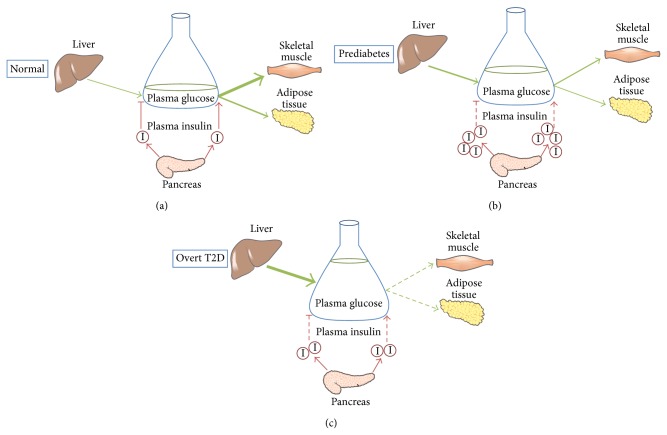
Under normal conditions, insulin is secreted from pancreatic *β*-cells in response to an increase in plasma glucose levels. It promotes glucose uptake into skeletal muscle and adipose tissue while suppressing hepatic glucose output, resulting in maintenance of blood glucose concentration to ~5 mM (a). In insulin resistant individuals, an increased amount of insulin is required to compensate for diminished effects on insulin-target organs, giving rise to hyperinsulinaemia. As insulin resistance worsens, blood glucose level gradually increases despite increased insulin secretion and a prediabetic state is established (b). In susceptible individuals, relative insulin deficiency progressively develops due to failure of *β*-cells to secrete adequate levels of insulin, resulting in loss of glucose homeostasis if exogenous insulin is not provided (c).

In healthy individuals, the variation of plasma glucose levels is kept minimal despite considerable fluctuations in nutrient intake (Figure 1(a)). The maintenance of circulating glucose levels under conditions of high nutrient availability is mainly mediated through the actions of insulin, a potent anabolic hormone secreted by the pancreatic *β*-cells in response to an increase in blood glucose level. Upon binding to its receptor, insulin initiates a cascade of downstream signaling events that influence a spectrum of enzymatic and transcriptional activities for the maintenance of glucose, lipid, and protein homeostasis [6]. Specifically, insulin promotes glucose uptake in skeletal muscles and adipose tissue by stimulating the membrane translocation of the GLUT4 transporter and activating enzymes involved in glycolysis [7]. In parallel, it facilitates carbohydrate disposal via both glycolysis and the nonoxidative pathways glycogen synthesis and* de novo* lipogenesis [8]. Meanwhile, insulin suppresses the processes generating circulating nutrients such as gluconeogenesis in the liver and lipolysis in the adipose tissue [6, 8]. The regulation of protein metabolism is another important aspect of insulin signaling, involving downregulation of proteolysis in skeletal muscles and promotion of protein synthesis through the mTOR pathway [9].

T2D is a pathological condition involving defects in both insulin action and secretion. It is characterised by elevations in postprandial and fasting blood glucose levels. Insulin resistance (IR), which is defined as the diminished biological effects of insulin on target tissues, is a major early defect in the pathogenesis of T2D [10, 11]. In the state of IR, the regulatory actions of insulin action on carbohydrate metabolism are impaired in target tissues. Accordingly, a state of hyperinsulinemia ensues due to the requirement of increased amounts of insulin to suppress hepatic glucose output from the liver and promote clearance of glucose into peripheral tissues (Figure 1(b)) [8]. When IR becomes more severe and glucose homeostasis cannot be maintained despite increased insulin levels, mild hyperglycemia sets in and a prediabetic state begins to manifest (Figure 1(b)). In susceptible individuals, the sustained increase in insulin secretion leads to the failure of pancreatic *β*-cells and the progression to T2D and marked hyperglycemia. In T2D patients this relative insulin deficiency necessitates insulin from exogenous sources to maintain whole-body glucose control (Figure 1(c)).

Obesity, especially the visceral form where mesenteric, epididymal, perirenal fat depots surround internal organs, is a well-recognised predisposing factor for developing T2D [12, 13]. Research in the last two decades has clearly demonstrated that, in the obese state, deposition of lipid in insulin-sensitive tissues such as muscle and liver is a key driver of IR [8]. In particular, bioactive lipid metabolites such as diacylglycerol and ceramide are thought to be the key culprits antagonising insulin action [8]. The ectopic accumulation of lipid metabolites is primarily due to elevated influx of fatty acids (FAs) into nonadipose tissues, due to the high availability of circulating FA coming from excess lipid intake and/or impaired insulin action to suppress adipose tissue lipolysis [8]. Liver steatosis is also secondarily enhanced by the paradoxical maintenance of insulin-stimulated* de novo* lipogenesis, despite reduced insulin sensitivity in glucose metabolism pathways [14]. In addition to inappropriate lipid deposition, obese individuals display chronic low-grade inflammation, especially in white adipose tissue, as well as elevations in local and systemic oxidative stress. Both of these factors are thought to attenuate insulin action, in part, by activating pathways that interfere with or oppose insulin signaling and thus they have also been implicated in the development of obesity-induced IR [15–17]. Collectively, the coexistence of obesity and T2D suggests that patients with these conditions have a fertile whole-body environment saturated in growth factor signals (insulin), an abundance of circulating nutrients (glucose, FAs), inflammatory cytokines, and reactive oxygen species.

## 3. Altered Metabolism in Cancer

Cancer is a heterogenous disease, characterised by the acquisition of successive mutations in protooncogenes and tumour suppressor genes [18]. These mutations allow tumour cells to sustain their growth signaling pathways, evade cell death, and continue to proliferate in an uncontrolled manner. Despite diversity in the precise molecular origin of different cancers, most (but not all) tumours tend to converge on a common metabolic phenotype, which was first described by Otto Warburg. In his seminal work in this field, Warburg observed that tumour cells exhibit aerobic glycolysis (i.e., high rates of glycolysis even in the presence of abundant oxygen) where pyruvate is converted to lactate instead of entering mitochondria for the TCA cycle [19, 20]. This phenomenon, known as the Warburg effect, has been the subject of intense research in recent years. Warburg reasoned that defective mitochondria prevent pyruvate from entering the TCA cycle and this underlies the enhanced rate of conversion of glucose to lactate [19]. However, later studies found that mitochondria in many tumour cell types are functional [21–23] and it is now clear that the alterations in the uptake and metabolism of different nutrients are critical for meeting both the bioenergetics needs of tumour cells and more importantly the increased requirement for biosynthesis of macromolecules.

**Figure 2:**
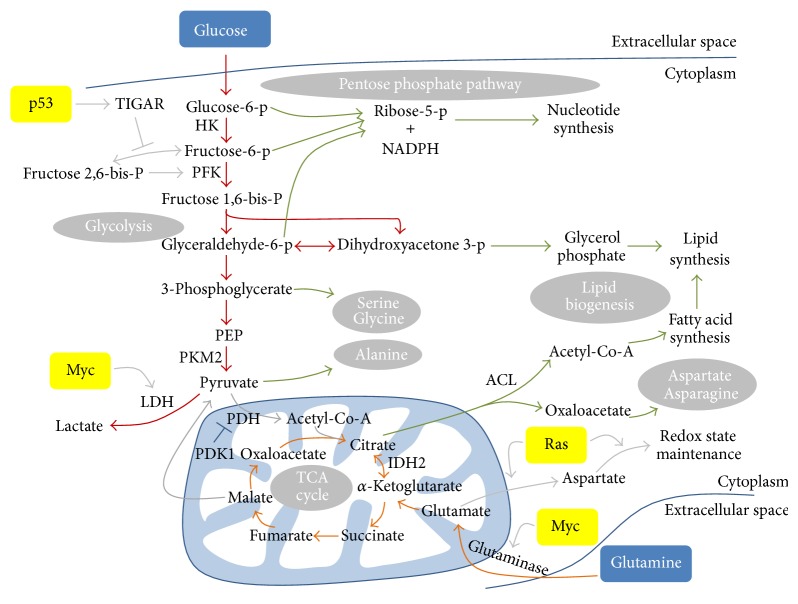
Glycolysis and glutaminolysis are two of the most important pathways for cancer cells. Increased glucose uptake, together with reduced glycolytic flux, accumulates glycolytic intermediates for synthesis of biomolecules such as nucleotides, amino acids, and lipids. Similarly, glutamine uptake is also increased. Glutamine is converted to glutamate by mitochondrial glutaminase. Glutamate is then converted to *α*-ketoglutarate which can be oxidised in the TCA cycle to generate ATP or reductively carboxylated to citrate. Citrate is exported to the cytoplasm where it is converted to acetyl-Co-A or oxaloacetate, which are used for synthesis of fatty acids or amino acids, respectively. Metabolic changes in cancer cells are driven by changes in the regulation of critical enzymes. Examples of these enzymes are shown in bold. Regulation of metabolic pathways by oncogenes (Myc and K-Ras) and tumour suppressor genes (p53) is also shown. Glycolysis is shown in red. Glutaminolysis is shown in orange. Biosynthetic pathways are shown in green. Other pathways are shown in grey.

Glucose and glutamine constitute two of the most important sources for meeting synthetic and energetic needs of tumour cells (Figure 2) [24]. The rate of glucose uptake in tumours is profoundly increased and the glycolytic intermediates provide building blocks for the synthesis of nucleotides, lipids, and amino acids [25–28]. Nucleotides are synthesised via the pentose phosphate pathway (PPP) using the intermediates generated by glycolysis including glucose 6-phosphate, fructose 6-phosphate, and glyceraldehyde 3-phosphate. The PPP also generates NADPH which is necessary for lipid synthesis and for maintenance of cellular redox potential. Lipid biogenesis also requires glycerol phosphate which is converted from another intermediate of glycolysis, dihydroxyacetone phosphate. Furthermore, the end product of glycolysis, pyruvate, is the substrate for synthesising nonessential amino acids alanine, whereas 3-phosphoglycerate is used for synthesising serine and glycine. The importance of aerobic glycolysis in cancer cells is highlighted by the fact that glucose withdrawal or inhibition of glucose uptake by small molecule inhibitors of PI3K signaling (discussed below) induces tumour cell death and tumour regression [29, 30] and that inhibition of lactate dehydrogenase (LDH) which converts pyruvate to lactate impairs cell proliferation [22, 31].

The increases in glucose uptake and glycolytic pathway activation are consequences of alterations in a range of metabolic enzymes and proteins. Tumour cells exhibit a marked increase in the expression of glucose transporters and their presence on the cell membrane, to achieve the required increase in glucose uptake [28, 32, 33]. This has been exploited in the clinic for the detection of tumours by imaging radioactive F-19-2-deoxyglucose uptake by positron emission tomography (PET). Once inside the cell, glucose is phosphorylated and trapped by hexokinase which is also hyperactivated in cancers. Cancer cells predominantly express the hexokinase II isoform [34], which is present on the outer membrane of mitochondria where it rapidly phosphorylates glucose. Despite the high rate of glucose uptake and phosphorylation, the overexpression of pyruvate kinase M2 (PKM2), which is less active than the M1 form in converting phosphoenolpyruvate (PEP) to pyruvate and subject to negative regulation by growth factor signaling, causes an overall reduction in glycolytic flux reaching the end-point pyruvate in cancer cells [26]. Overall, increased glucose uptake and reduced glycolytic flux going to completion result in the accumulation and channeling of glycolytic intermediates towards biosynthetic pathways.

Due to the diversion of most glucose-derived intermediates to biosynthesis, glutamine uptake is also increased in tumours to replenish the depletion of TCA cycle intermediates which are normally supplied from glucose sources and to fuel mitochondrial ATP production. Additionally, recent work has shown that under certain conditions glutamine can also play another important role in the growth of tumour cells, providing acetyl-CoA for lipid synthesis through a process known as reductive carboxylation [35–38]. Similar to glucose, the expression of membrane glutamine transporters (e.g., ASCT2), in particular the high affinity isoforms, is elevated in cancer [39]. Furthermore glutaminase, the enzyme responsible for the metabolism of glutamine, is markedly increased in many cancers, consistent with an addiction of tumours to the use of this nutrient [40].

The biosynthesis of lipids is another key aspect of the tumour metabolic program. Cancer cells perform* de novo* fatty acid synthesis extensively from glucose and glutamine-derived precursors and NADPH to supply materials for the production of membranes and signaling molecules, as opposed to the majority of normal cells that rely mainly on lipids from the environment [41]. Several proteins involved in lipogenesis including ATP citrate lyase (ACL) [42], acetyl-CoA carboxylase (ACC) [43], fatty acid synthase (FAS) [44], and sterol response element binding protein (SREBP) [45] have been shown to be intimately related to cancer cell growth and survival. In addition, a subset of cancers also scavenges lipids from adipocytes [46] and the circulation [47] by upregulating FA transporters fatty acid binding protein 4 and CD36. Together, the coordinated upregulations of aerobic glycolysis, glutamine uptake, and biosynthetic processes represent a fundamental shift in cellular metabolic landscape to support tumour growth and rapid expansion.

## 4. Factors Driving Metabolic Changes in Cancer

A major factor leading to alterations in metabolic enzymes and pathways in cancer is the presence of tumour hypoxia. Hypoxia leads to the stabilisation of hypoxia-inducible factors 1 and 2 (HIF-1 and HIF-2) and HIF-1 is known to upregulate 9 out of 10 enzymes of glycolysis [48, 49]. HIF-1 also prevents entry of pyruvate into the TCA cycle, firstly by upregulating LDH which converts pyruvate to lactate and secondly by upregulating pyruvate dehydrogenase kinase (PDK1) which inhibits PDH thus blocking the conversion of pyruvate to acetyl-CoA [50, 51]. HIF-2, on the other hand, increases Myc function (discussed below) allowing cells to proliferate under hypoxia [52]. HIF transcription factors themselves are under regulation of the TCA cycle enzymes succinate dehydrogenase (SDH) and fumarate hydratase (FH). Although mitochondria are not typically defective in tumour cells, SDH and FH enzymes are mutated in some types of cancers [53]. Mutations in these enzymes cause an accumulation of fumarate and succinate, which results in the inhibition of prolyl-hydroxylases that mediate degradation of HIF proteins, thereby enhancing glycolysis [53].

The changes in metabolic enzymes in cancer are not always an adaptation to hypoxia, as cancers such as leukaemia and lung cancer have abundant oxygen supply during tumourigenesis but still operate aerobic glycolysis [54–56]. There is increasing evidence that oncogenes and tumour suppressor genes directly regulate metabolic pathways in tumourigenesis. Not only do mutations in these genes reprogram metabolic pathways for progression of the tumours, but also metabolic changes induced by them may be primary events in cellular transformations [57]. Myc, an oncogene frequently mutated in many cancers, was one of the first to be linked to metabolism, as it directly activates expression of LDH [31, 58]. Myc target genes include enzymes of glycolysis, glutaminolysis, and fatty acid synthesis [39, 59, 60]. The enhanced expressions of membrane glutamine transporters and mitochondrial glutaminase and the consequent increase in glutaminolysis are mediated by the Myc oncogene [39, 40]. Conversely, inhibition of mitochondrial glutaminase by pharmacological inhibitors impairs tumour growth of Myc-expressing B cells in xenograft models [61]. Similarly, glutamine removal from culture media results in cell death in Myc overexpressing cancer cells [62].

Another well characterised oncogene, Ras, can also promote changes favouring tumour growth and proliferation. For example, oncogenic K-Ras, which is associated with over 90% of pancreatic ductal adenocarcinoma (PDAC), mediates changes in both glucose and glutamine metabolism that are essential for PDAC maintenance. K-Ras stimulates glycolytic flux and diverts glycolytic intermediates to hexosamine biosynthesis pathway (HBP) and PPP [63]. This effect appears to be dependent on Myc, as its knockdown significantly downregulated the expression of metabolic genes involved in glycolysis, HBP and PPP [63]. PDAC cells also operate a distinct metabolic pathway for glutamine metabolism where glutamine is metabolized through the noncanonical pathway to produce aspartate which is subsequently transported to the cytoplasm for the maintenance of NADPH/NADP^+^ ratio and cellular redox state [64]. Downregulation of enzymes in this pathway leads to suppression of PDAC growth* in vitro* and* in vivo* [64].

Mutation of tumour suppressor genes, such as p53, is a critical event in many cancers, but their emerging roles in metabolism have been elucidated only recently [65]. One of the most discussed links between p53 and metabolism is via TIGAR-dependent inhibition of glycolysis [25]. The p53 target gene TIGAR lowers intracellular concentration of fructose 2,6-bisphosphate (FBP), an allosteric activator of phosphofructokinase, thus inhibiting glycolysis and diverting glucose to PPP. Additionally, p53 represses transcription of glucose transporters GLUT1 and GLUT4 [66]. Apart from suppressing glycolysis, p53 also influences mitochondrial metabolism by increasing the transcription of synthesis of cytochrome c oxidase 2 (SCO2) which assembles into oxidative phosphorylation complex and enhances mitochondrial respiration [67]. Therefore, loss of tumour suppressors confers growth advantage to tumours from a metabolic angle, by favouring a metabolic profile conductive to rapid cell proliferation.

## 5. Metabolic Changes in Diabetes Can Facilitate Tumourigenesis

As noted above, epidemiological evidence shows that individuals with diabetes have significantly higher likelihood of developing multiple types of cancers [3]. Amongst these, organs associated with energy metabolism such as liver and pancreas have the strongest association with diabetes. Furthermore, diabetic patients with colorectal, breast, and endometrial cancers have significantly higher chances of dying of cancer than normal individuals [68]. The mechanisms driving cancers in diabetic patients are still not entirely clear, but some possibilities are discussed below.

In early stages of diabetes, pancreatic *β*-cells produce excess amount of insulin, resulting in hyperinsulinemia. While insulin-target organs are resistant to the actions of insulin in diabetes, hyperinsulinemia may have progrowth effects on a nascent tumour by allowing the tumour to overcome an important early barrier in tumourigenesis, that is, lack of growth factor signaling. There is epidemiological data to suggest that insulin secretion rate and insulin-like growth factor 1 (IGFI) levels influence cancer risk and/or cancer progression [69, 70]. Insulin and IGFI stimulate the proliferation of tumour cells* in vitro* [71] and promote glucose uptake in the subset of tumours that are insulin-dependent [72, 73]. The IGFI receptor (IGF1R) is necessary for the transforming ability of several oncogenes, suggesting that parallel growth factor signaling-mediated metabolic changes are crucial for cellular transformation [74]. In line with the above observations, reduced growth factor signaling leads to decreased tumour growth in mouse models [69]. The above observations indicate that hyperinsulinemia or administration of synthetic insulin in diabetes may enhance growth factor signaling and promote glucose usage to promote tumour growth. As many tumours devise means to evade regulations of growth factor signaling, we propose that insulin may serve as the spark to initiate cancer development at early stages when self-sufficiency of growth factors has not yet been established.

Hyperglycemia, another characterising feature of diabetes, may also contribute to enhanced cancer risk [75]. Given the central role that glycolysis plays in tumour development, elevated glucose levels in the circulation are likely to provide abundant glucose resources and a concentration gradient for convenient usage by cancer cells. Indeed, epidemiological evidence suggests that hyperglycemia in cancer patients contributes to increased likelihood of tumour recurrence, metastasis, or fatal outcome compared to patients with hyperglycemia [75]. Additionally to the direct metabolic role, hyperglycemia in a subset of tumour cells can lead to increased production of ROS from mitochondrial respiration, which below certain levels can lead to DNA damage that are not severe enough to induce apoptosis [75, 76] but may give rise to mutations in protooncogenes and tumour suppressor genes or other changes that are beneficial for the tumour. For example, hyperglycemia-related increased ROS production in pancreatic cancer cell lines such as Panc-1 and BxPC-3 increases cell motility and invasiveness, indicating hyperglycemia may contribute to pancreatic cancer metastasis [9]. Enhanced glucose metabolism may also prevent cytochrome c mediated-cell death in cancer cells [77] and confer resistance to chemotherapy [78, 79], both favouring continued tumour growth.

At the systemic level in diabetes, the excess availability of nutrients and local changes in tissues, including adipose tissue, leads to chronic low-grade inflammation. For instance, the levels of cytokines such as TNF*α* and IL-6 are increased, as a result of both the stimulation of monocytes and macrophages by excess nutrients and the increased expression and release from inflamed adipose tissue [80–83]. Inflammation is important in tumourigenesis as it contributes to all stages of tumourigenesis, including angiogenesis and metastasis [84]. Both TNF*α* and IL-6 have been shown to promote tumour invasiveness and metastasis by secretion of matrix remodelling proteins matrix metalloproteinases [85]. IL-6-deficient mice are resistant to multiple myeloma, while neutralization of TNF*α* switches inflammation-driven metastatic growth to inflammation-induced tumour regression [86–88]. Thus, diabetes associated hyperglycemia and hyperlipidaemia can promote tumourigenesis by inducing inflammation.

There may also be a more direct link between the obesity that is commonly observed in diabetes and the development of some tumours. Recent work has shown that there is cross talk between adipocytes and certain types of tumours, whereby signals from tumours can lead to enhanced provision of FA from the surrounding adipocytes for use in energy production [46]. The generality of this mechanism for tumours that exist in regions with high levels of adipocytes (e.g., breast) remains to be elucidated.

## 6. Treatments for Diabetes Can Impact Cancer Progression

There are a range of glucose-lowering therapeutic agents currently prescribed for T2D. The most widely used front-line drug is metformin, which alters intracellular metabolism in insulin-target tissues (liver, skeletal muscle, and adipose tissue) to reduce end-organ resistance to the actions of insulin [89]. Other therapies are designed to increase endogenous insulin secretion by directly acting on pancreatic *β*-cells (sulfonylurea) or by enhancing the action of insulin secretion-promoting gut peptides (incretin mimetics) [10, 89]. At late stage of T2D, relative insulin deficiency due to heightened IR and pancreatic *β*-cell failure makes the administration of exogenous insulin a necessity. As noted above, high level of circulating insulin may facilitate cancer propagation, and thus insulin secretagogues and exogenous insulin are likely to increase cancer risk. Metformin, on the other hand, has been observed to reduce incidence and mortality of several cancer types compared to other diabetes medications, based on numerous population-based epidemiological studies and meta-analysis [90–93].

There are multiple aspects of diabetes that are improved by metformin, including suppression of hepatic overproduction of glucose and improvement of peripheral insulin sensitivity. One mechanism by which metformin achieves these effects is through activation of the energy sensor AMP-dependent protein kinase (AMPK). AMPK has versatile functions in the regulation of cellular energy metabolism, some of which overlap with and enhance the effects of insulin, such as the augmentation of glucose uptake in peripheral tissues [94]. Furthermore, AMPK inhibits endogenous lipid synthesis and promotes fatty acid oxidation, contributing to diminished lipid storage in nonadipose tissues and improved insulin sensitivity [95]. The way that metformin activates AMPK is thought to be through alterations in nucleotide levels. As a positively charged drug, metformin is taken into the mitochondrial matrix due to the inner membrane electrical gradient where it inhibits complex I of the respiratory chain in a time-dependent and self-limiting manner [96, 97]. The blockage of mitochondrial energy production through oxidative phosphorylation leads to changes in the AMP/ATP and ADP/ATP ratios, which signal energetic crisis that activates AMPK. Independent of its effects to activate AMPK it was recently shown that metformin suppresses hepatic glucose production by restraining glucagon-dependent gluconeogenesis [98].

With respect to cancer, several mechanisms have been proposed to underlie the beneficial antitumour effects of metformin and the more potent member of the biguanide class of drugs, phenformin. Given the tumour-promoting roles of plasma insulin and glucose, the alleviation of IR in insulin-target organs and the resulting reduction in glucose and insulin concentration in the circulation likely contribute to metformin-mediated tumour-suppressive effects in diabetic patients [99]. In addition, the accumulation of biomass in neoplastic cells is attenuated by metformin, which inhibits mTOR (mammalian target of rapamycin) signaling via Rag and Rac1 GTPase [100, 101].

The more well-described mechanism proposed to mediate the effects of metformin is activation of AMPK, which as noted above reprograms nutrient metabolism in response to energetic stress, favouring catabolic over anabolic pathways. AMPK signaling is downregulated in breast and ovarian cancers [102, 103] and its upstream activator LKB1, a well-known tumour suppressor, is nonfunctional in subsets of endometrial and lung cancers [104, 105]. Activation of AMPK through metformin treatment inhibits breast cancer growth through inducing cell-cycle arrest and opposing protein synthesis [106, 107]. Metformin-induced activation of AMPK is also associated with reduced growth of a number of other tumour types [108, 109]. Many of the effects of metformin are also seen when tumour cells are treated with the AMPK activator AICAR, which promotes oxidative metabolism and favours lipid utilization [110]. To further substantiate the tumour-suppressive role of AMPK in opposing cancer-related metabolic alterations, Faubert et al. showed that inactivation of the AMPK *α* catalytic subunit in both transformed lymphoma cells and nontransformed counterparts resulted in a shift towards aerobic glycolysis, increased incorporation of glucose-derived carbons into lipids, and biomass production while mice deficient in AMPK*α* had accelerated rate of lymphomagenesis [111]. Collectively these studies highlight the important role AMPK likely plays in the efficacy of metformin and suggest that the development of agents mimicking some of the effects (e.g., inhibition of lipogenesis and promotion of fat oxidation) of AMPK activators may have therapeutic relevance.

In 2004, Shaw and colleagues reported the paradoxical observation that tumour suppressor LKB1-deficient mammalian cells are resistant to oncogene-induced transformation but more prone to apoptotic cell death in response to cellular energy stress [112]. This intriguing finding raises the possibility that oblation of the energy-sensing LKB1-AMPK axis, while conferring biosynthetic and proliferative advantages, also imparts vulnerability to the cells so that they are hypersensitive to energetic crisis-induced killing. Indeed, non-small cell lung cancer (NSCLC) mice harbouring Kras and Lkb1 mutations, compared to those with Kras and p53 mutations, are selectively targeted by phenformin, leading to prolonging of survival [113]. Another potential application of biguanides as cancer-metabolism based therapies could be for tumours that have greater reliance on mitochondrial oxidative metabolism rather than the classical aerobic glycolysis. For example, a subset of human melanoma tumours was recently characterized to overexpress the master regulator of mitochondrial biogenesis PGC1*α* and exhibit increased mitochondrial energy metabolism [114]. For negative PGC1*α* melanoma cells, it was demonstrated that inhibition of BRAF, the most frequently overexpressed oncogene in melanoma, switched on a mitochondrial phosphorylation gene program including PGC1*α* and rendered the cells addicted to oxidative metabolism for a window of period before resistance developed [115]. A separate study reported synergistic tumour-suppressive effects of combining phenformin and a BRAF inhibitor in melanoma on attenuating mTOR signaling and inducing apoptosis, which were attributed to cross talk between AMPK and BRAF signaling pathways [116]. It is unknown if the cooperation between BRAF inhibition and phenformin also acts via the induction of addiction to oxidative phosphorylation by the former and inhibition of mitochondrial respiratory chain complex I by the latter, but if the idea of synthetic lethality involving biguanide can be generalised to other cancer types, the impact on rational therapeutic design will be considerable.

Another prominent class of diabetic drug is the thiazolidinediones (TZDs) including pioglitazone and rosiglitazone. TZDs are agonists of peroxisome proliferator-activated receptor *γ* (PPAR*γ*) which are predominantly expressed in adipose tissue [117]. They function primarily by inducing adipocyte proliferation and increasing adipose tissue lipid storage capacity to reduce fatty acid overflow to ectopic sites such as muscle, liver, and pancreas, along with exerting transcriptional control of genes involved in glucose and lipid metabolism [117]. TZDs have been shown to induce cell-cycle arrest, apoptosis, differentiation, and metastasis in a range of* in vitro* and* in vivo* cancer models [118]. Interestingly, some of the anticancer effects such as inhibitions of cell-cycle progression and invasiveness have been suggested to be independent of PPAR*γ* activation [119, 120]. Despite these* in vitro* effects, epidemiological studies and meta-analysis over the past few years investigating the association between TZD use and cancer risk generated mixed results with the overall conclusion that TZDs reduce or do not affect the incidence of most cancer types but may increase the likelihood of developing bladder cancer [121–126]. The mechanisms responsible for these disparate findings are still under investigation.

## 7. Conclusions and Future Perspectives

T2D is increasing in prevalence across the world and with it comes the well-described complications, as well as an increased risk of many other diseases (e.g., cardiovascular disease). There is growing evidence that diabetes can also increase the risk of certain types of cancers. This relationship is not fully understood and there are many unanswered questions. For example, what are the exact features of diabetes that promote these types of cancer? What is the relative importance of different circulating nutrients, given the high level of glucose and lipids in diabetes and the recently described branched-chain amino acid signature [127]? Why does diabetes only increase the risk of certain types of cancers, but not all of them? Since compounds such as metformin appear to be beneficial for both T2D and cancer, we suggest that developing further compounds with dual effectiveness in both diseases, along with the pursuit of the unresolved questions above, should be the focus of future research in this area.
